# A systematic review of cognitive decline in dementia with Lewy bodies versus Alzheimer’s disease

**DOI:** 10.1186/s13195-014-0053-6

**Published:** 2014-09-16

**Authors:** Monica H Breitve, Luiza J Chwiszczuk, Minna J Hynninen, Arvid Rongve, Kolbjørn Brønnick, Carmen Janvin, Dag Aarsland

**Affiliations:** 1 Section of Mental Health Research, Clinic of Psychiatry, Helse-Fonna HF Haugesund Hospital, Haugesund, 5504, Norway; 2 Old Age Department, Clinic of Psychiatry, Helse-Fonna HF Haugesund Hospital, Haugesund, 5504, Norway; 3 Neurological Department, Clinic of Medicine, Helse-Fonna HF Haugesund Hospital, Haugesund, 5504, Norway; 4 Department of Clinical Psychology, University of Bergen, Christies gate 12, Bergen, 5015, Norway; 5 NKS Olaviken Hospital for Old Age Psychiatry, Ulriksdal 8, Bergen, 5009, Norway; 6 Faculty of Medicine, University of Bergen, Bergen, 5020, Norway; 7 TIPS, Regional Centre for Clinical Research in Psychosis, Stavanger University Hospital, Stavanger, 4068, Norway; 8 Network for Medical Sciences, Stavanger University Hospital, Stavanger, 4068, Norway; 9 Centre for Age Related Medicine, Stavanger University Hospital, Stavanger, 4068, Norway; 10 Department NVS, Center for Alzheimer Research, Division for Neurogeriatrics, Karolinska Institutet, Huddinge, 14157, Sweden

## Abstract

**Introduction:**

The aim of this review was to investigate whether there is a faster cognitive decline in dementia with Lewy bodies (DLB) than in Alzheimer’s disease (AD) over time.

**Methods:**

PsycINFO and Medline were searched from 1946 to February 2013. A quality rating from 1 to 15 (best) was applied to the included studies. A quantitative meta-analysis was done on studies with mini mental state examination (MMSE) as the outcome measure.

**Results:**

A total of 18 studies were included. Of these, six (36%) reported significant differences in the rate of cognitive decline. Three studies reported a faster cognitive decline on MMSE in patients with mixed DLB and AD compared to pure forms, whereas two studies reported a faster decline on delayed recall and recognition in AD and one in DLB on verbal fluency. Mean quality scores for studies that did or did not differ were not significantly different. Six studies reported MMSE scores and were included in the meta-analysis, which showed no significant difference in annual decline on MMSE between DLB (mean 3.4) and AD (mean 3.3).

**Conclusions:**

Our findings do not support the hypothesis of a faster rate of cognitive decline in DLB compared to AD. Future studies should apply recent diagnostic criteria, as well as extensive diagnostic evaluation and ideally autopsy diagnosis. Studies with large enough samples, detailed cognitive tests, at least two years follow up and multivariate statistical analysis are also needed.

## 1 Introduction

Dementia with Lewy bodies (DLB) and Alzheimer’s disease (AD) are the two most common subtypes of neurodegenerative dementia, representing 15 to 20% and 65% of all dementia cases, respectively [1]. DLB is characterized clinically by symptoms such as visual hallucinations, Parkinsonism and fluctuating cognition in addition to cognitive impairment with typically more visuospatial and executive impairment relative to memory impairment [2]. There is some evidence that DLB patients have more rapidly progressing dementia compared to AD [3], and more recent studies also reported a more severe course with shorter survival [4], higher rate of nursing home admissions [5] and higher costs in DLB as compared to AD [6].

An overlap in neuropathology between AD and DLB has been noted [7]. Parkinson’s disease (PD) and DLB also share some clinical and pathological features [8]. Subgroups with different cognitive profiles have been described in patients with PD [9], and there is evidence that this differentiation is related to the rate of cognitive decline [10]. Similar neuropsychologically defined subgroups may exist also in DLB [8], which could also predict differences in the rate of progression to end-stage dementia. Data supports accelerated disease progression when AD and DLB pathologies are present together [11].

To our knowledge, no systematic review has compared rate of cognitive decline in DLB versus AD. We therefore systematically reviewed the literature to find studies assessing overall cognitive decline in DLB and AD. We specifically noted studies that had investigated the potential differences in cognitive decline in subgroups with DLB and the effect of employing different diagnostic criteria.

## 2 Methods

PsycINFO and Medline were searched in February 2013, using key words listed in Table 1. References from reviewed articles were also searched for relevant studies. The following inclusion criteria were used: a) paper published in a peer-reviewed journal; b) written in English; c) DLB or mixed AD/DLB compared with AD; d) application of at least one neuropsychological test, and e) at least 6 months follow up. The following exclusion criteria were used: a) drug trials, and b) survival studies with death as the only outcome.

**Table 1:** Search history

	**Medline**	**PsycINFO**	
	**(1946 to February 2013)**	**(1806 to February 2013)**	
**Key words**	Alzheimer’s disease and Lewy body disease, or Lewy bodies	Alzheimer’s disease and dementia with Lewy bodies	
**Key words**	Neuropsychology, or neuropsychological tests, or Cognition, or cognition disorders	Neuropsychology, or neuropsychological assessment, or neuropsychological assessment, or Cognition, or cognitive impairment, or	
**Key words**	Disease progression, or longitudinal studies	Disease course, or disease prognosis, or longitudinal studies	
**Search results**	70	97	
**Included**			18

### 2.1 Quality assessment

Two independent raters rated all studies with a self-designed quality scale and arrived at the same result. The domains, a) number of patients included; b) follow-up time; c) clinical criteria; d) autopsy, and e) neuropsychological tests) were rated on a four-point scale adapted from Aarsland *et al*. (2005) [12]: 0 (none), 1 (poor), 2 (fair) and 3 (good). See Table 2. Studies could be assigned 1 to 15 points.

**Table 2:** Quality assessment criteria

	**Score**			
	**3**	**2**	**1**	**0**
**Patients at baseline, number**	>151	101 to 150	51 to 100	<50
**Follow-up time, years**	>3 or mean ≥3	3	2	≤1
**Clinical criteria**	Established criteria for AD + DLB criteria from 2005	Established criteria for AD + DLB criteria from 1992 or 1996	Used criteria for one type of dementia	No criteria used
**Autopsy, % of participants**	100	>50	>25	None

AD, Alzheimer’s disease; DLB, dementia with Lewy bodies; BNT, Boston naming test; CERAD, Consortium to Establish a Registry for Alzheimer’s Disease evaluation; DRS, dementia rating scale; ESD, extended scale for dementia; HVLT-R Hopkins verbal learning test-revised; mMMS, modified mini-mental state examination; MMSE, mini mental state examination; MTS, 37 item mental test score.

### 2.2 Statistical analysis

For studies reporting mini mental state examination (MMSE) results, standardized mean difference in annual progression between DLB and AD was calculated as the difference between annual progression between the DLB and AD groups divided by the pooled standard deviation across groups in each included study. The standardized mean differences were combined in a random-effects model to obtain summary estimates of the effect in each study. The overall results from each trial were then combined using a random-effects model to obtain a pooled summary estimate of effect across all trials [13]. To assess heterogeneity, the *I*^2^ as proposed by Higgins and colleagues [14] was chosen, indicating the percentage of total variation across studies due to heterogeneity.

## 3 Results

Of the 18 studies included in this review (see Table 3), six (36%) reported a statistically significant difference in cognitive decline over time between AD and DLB (see Table 4). Three studies reported a faster cognitive decline on cognitive screening tests in the neuropathologically mixed AD/DLB group [3],[15],[16] compared to those with pure AD or DLB. One study reported a faster decline in DLB than in AD on verbal fluency [17], and two in AD compared to DLB on memory [18],[19]. For a full description of neuropsychological tests used in included studies, see Table 3.

**Table 3:** Study characteristics and main findings of included studies

**Study**	**Sample, male/female ratio (m/f), mean age (SD)**	**Follow-up period**	**Neuropsychological tests**	**AD versus DLB comparison**	**Test scores, mean (SD)**
**McKeith** ***et al*., 1992 [**[20]**]**	AD 37	Baseline and late stage	MTS	No significant difference	MTS baseline
	m/f 13/24				AD 15.9 (1.8)
	y 74.7 (0.9)				SDLT 24.5 (1.7)
	SDLT 21				MTS late stage
	m/f 12/9				AD 9.3 (2.1)
	y 73.3 (1.6)				SDLT 18.2 (2.3)
**Ballard** ***et al*., 1996 [**[17]**]**	AD 53	1 y	CAMCOG	SDLT faster decline of verbal fluency	Scores for subtests n/a
	m/f, n/a				
	Y, n/a				CAMCOG total, baseline
	SDLT 7				AD 42.7 (17.9)
					SDLT 47.7 (18.0)
	m/f, n/a				CAMCOG mean annual decline
	Y, n/a				
	VaD 14				AD 13.2 (12.6)
	m/f, n/a				SDLT 27.0 (19.8)
	Y, n/a				
**Ballard** ***et al*., 1998 [**[21]**]**	AD 30	1 y	MMSE	No significant difference	MMSE baseline
	m/f 9/21				AD 13.9
					DLB 14.9
	y 81.7				MMSE mean annual decline
	DLB 42				
					AD 4.1
	m/f 19/24				DLB 3.9
	y 73.6				
**Olichney** ***et al*., 1998 [**[3]**]**	AD 148	Mean 3 y	MMSE	LBV faster decline	MMSE baseline
	m/f 80/68				
	y 74.0 (7.9)				AD 17.8 (6.0)
	LBV 40				LBV 18.2 (5.5)
	m/f 25/15				MMSE 1 y (n = 136/35)
	y 72.4 (6.5)				
					AD 14.3 (7.2)
					LBV 12.5 (7.5)
					MMSE 2 y (n = 93/17)
					AD 12.3 (7.9)
					LBV 8.1 (6.3)
					MMSE 3 y (n = 59/12)
					AD 10.1 (8.4)
					LBV 4.5 (6.5)
					MMSE 4 y (n = 35/4)
					AD 9.1 (7.9)
					LBV 2.5 (3.0)
					MMSE mean annual decline
					AD 4.1 (3.0)
					LBV 5.8 (4.5)
**Heyman** ***et al*., 1999 [**[18]**]**	AD 74	Annual controls	CERAD (including CDT, calculation test, serial subtraction, CDR, BNT, MMSE, 10-item word list memory, recall and recognition, constructional praxis, two of the six items of the orientation-memory-concentration test)	AD faster decline in delayed recall	32% of LBV versus 15% of AD remembered any item on word list recall at last evaluation
	m/f 47/27				
	y 41% >74 y				
	AD/LBV 27				
	m/f 14/13				
	y 37% >74 y				
**Lopez** ***et al*., 2000 [**[22]**]**	AD 98	Mean 59 months	MMSE	No significant difference	MMSE baseline
	m/f 50/48				
	y 70.8 (9.4)				AD 16.0 (6.5)
	AD/DLB 44				AD/DLB 16.2 (5.1)
	m/f 20/24				
	y 72.3 (6.0)				
**Stern** ***et al*., 2001 [**[23]**]**	AD 32	Annual controls, longest 9.9 y	mMMSE (including WAIS-R digit span forward, backward, attention, calculation, general knowledge, language, construction), CDR	No significant difference	mMMSE baseline
	m/f 16/16				AD 36.7 (6.3)
	y 73.0 (9.0)				LBV 37.3 (6.2)
	LBV 19				mMMSE mean annual decline 3.6 (both groups)
	m/f 17/2				
	y 73.6 (6.8)				
**Ballard** ***et al*., 2001 [**[24]**]**	AD 101	1y	MMSE, CAMCOG	No significant difference	MMSE n = 203
	m/f 30/71				MMSE baseline
	probable AD 61 m/f 17/44				prob AD 17.7 (5.1)
					poss AD 17.2 (5.2)
	y 81.9 (4.8)				
					DLB 15.6 (7.0)
	possible AD 40				
					MMSE mean annual decline
	m/f 13/27				
	y 79.0 (7.8)				AD 4.9 (3.6)
	DLB 64				DLB 4.3 (4.2)
	m/f 26/38				CAMCOG n = 154
					Baseline 57.5 (18.8)
	y 76.6 (7.7)				
	VaD 38				CAMCOG mean annual decline
	m/f 22/16				
	y 76.8 (7.7)				Probable AD 15.0 (10.1)
					Possible AD 14.4 (9.8)
					DLB 11.9 (12.2)
**Helmes** ***et al*., 2003 [**[25]**]**	AD 15	50 months	ESD	No significant difference	Scores n/a
	m/f 9/6				
	y 70.3 (7.6)				
	AD/DLB 8				
	m/f 5/3				
	y 69.3 (11.2)				
	DLB 7				
	m/f 5/2				
	y 69.1 (4.1)				
**Johnson** ***et al*., 2005 [**[26]**]**	AD 66	Annual controls,	WMS (digits forward, backward, logical memory and associate learning), BVRT, word fluency, BNT, WAIS (Digit Symbol and Block Design), TMT A, Crossing Off, CDR	No significant difference	Follow-up scores n/a. For baseline scores for all tests see article
	m/f 39/27	1 to 20 assessments			
	y 77.0 (8.1)				
	AD/DLB 57				
	m/f 31/26				
	y 75.2 (9.7)				
	DLB 9				
	m/f 8/1, age 72.6 (5.7)				
**Kraybill** ***et al*., 2005 [**[15]**]**	AD 48	Annual controls	MMSE, DRS	AD/LBP faster decline than AD and LBP	MMSE baseline
	m/f 18/30				AD 20.6 (3.9)
	y at onset 77.5				AD/LBP 20.7 (3.7)
	(7.34)				LBP 20.7 (3.8)
	AD/LBP 65				MMSE mean annual decline
	m/f 24/41				AD 3.5 (0.4)
	y at onset 74.8 (6.6)				AD/LBP 5.0 (0.5)
					LBP 3.4 (0.7)
	LBP 22				DRS baseline
	m/f 16/6				AD 114.7 (2.1)
	y at onset 76.5 (5.3)				AD/LBP 114.2 (1.8)
					LBP 114.2 (2.7)
					DRS mean annual decline
					AD 9.6 (1.5)
					AD/LBP 15.3 (1.9)
					LBP 8.8 (1.7)
**Stavitsky** ***et al*., 2006 [**[19]**]**	AD 55	Mean 3 y	mMMSE (incl WAIS-R digit Span forward, backward, attention, calculation, general knowledge, language, construction), HVLT-R	AD faster decline on recognition.	mMMSE baseline
	m/f 21/34				AD 39.0 (7.6)
					DLB 38.1 (8.3)
	y 73.1 (8.3)				HVLT-R n/a
	DLB 28				
	m/f 19/9				
	y 73.5 (7.6)				
**Williams** ***et al*., 2006 [**[27]**]**	AD 252	< 5 y	MMSE, CDR, WMS (mental control, logical memory, digit span forward and backward, associate learning), BVRT, WAIS (information, digit symbol, block design), word fluency, BNT, Crossing off, TMT A	No significant difference.	Scores n/a
	m/f 95/157				
	y 77.8 (9.5)				
	DLB 63				
	m/f 38/25				
	y 73.5 (8.7)				
**Hamilton** ***et al*., 2008 [**[28]**]**	AD 44	2 y	DRS, WISC-R (block design), CDT copy, BNT	Poor baseline visuospatial skills (block design <20, CDT copy <3) were strongly associated with faster decline in DLB, but not AD.	DRS baseline
	m/f 20/24				AD 114.4 (15.4)
	y 72.0 (5.6)				DLB 109.5 (11.4)
	DLB 22				DRS 1 y mean decline
	m/f 14/8				
	y 73.4 (6.2)				AD 7.9 (11.6)
					DLB 17 (24.2)
					DRS 2 y mean decline
					AD 23.9 (24.7)
					DLB 39.3 (35.1)
					Other scores n/a
**Hanyu** ***et al*., 2009 [**[29]**]**	AD 111	5 y	MMSE	No significant difference	MMSE
	m/f 37/74				Baseline n = 111/56
	y 77.5 (6.2)				AD 20.3 (3.7)
	DLB 56				DLB 20.7 (3.8)
	m/f 30/26				1 y n = 111/56
	y 78.1 (5.2)				AD 19.4 (4.8)
					DLB 20.5 (4.2)
					2 y n = 102/40
					AD 17.7 (5.2)
					DLB 18.0 (4.8)
					3 y n = 72/25
					AD 16.2 (5.0)
					DLB 17.0 (5.3)
					4 y n = 51/19
					AD 14.2 (4.5)
					DLB 13.4 (4.0)
					5 y n = 16/5
					AD 11.4 (5.2)
					DLB 10.6 (4.0)
**Nelson** ***et al*., 2009 [**[16]**]**	AD 107	Mean 4 y	MMSE	AD/DLB had a faster decline than DLB and AD.	MMSE baseline n/a
	m/f n/a				MMSE final
	y n/a				AD 10.7 (8.6)
	AD/DLB 27				AD/DLB 10.6 (8.6)
	m/f n/a				DLB 15.6 (8.7)
	y n/a				
	DLB 9				
	m/f n/a				
	y n/a				
**Wood** ***et al*., 2012**[30]	AD 16	1 y	MMSE, CAMCOG, NEVIP	No significant difference.	MMSE baseline
	m/f 12/4				AD 21.3 (3.2)
	y 78.9 (6.1)				DLB 24.5 (3.3)
	DLB 10				MMSE decline from baseline
	m/f 9/1				
	y 78.2 (7.4).				AD 2.1 (3.6)
	Controls 28				DLB 1.8 (3.1)
	m/f 16/12				CAMCOG baseline
	y 79.5				AD 71.4 (9.7)
					DLB 79.1 (12.0)
					CAMCOG decline from baseline
					AD 7.4 (10.7)
					DLB 4.3 (7.3)
**Walker** ***et al*., 2012**[31]	AD 100	1 y	MMSE, CAMCOG-R, VOSP, CDR	No significant difference.	MMSE baseline
	m/f 48/52				AD 21.5 (4.5)
	y 74,9				DLB 21.4 (3.9)
	DLB 58				MMSE follow up (n = 81/33)
	m/f 37/21				AD 19.0 (6.2)
	y 74,2				
					DLB 18.5 (6.0)
					CAMCOG-R baseline
					AD 66.3 (15.6)
					DLB 66.0 (13.5)
					CAMCOG-R follow up
					(n = 81/33)
					AD 59.6 (20.3)
					DLB 56.3 (19.7)

AD, Alzheimer’s disease; DLB, dementia with Lewy bodies; LBP, Lewy body pathology; LBV, Lewy body variant; n/a, not available; SDLT, senile dementia of Lewy body type; VaD, vascular dementia; y, years; BNT, Boston naming test; BVRT, Benton visual retention test; CAMCOG, Cambridge cognitive examination; CAMCOG-R, Cambridge cognitive examination-revised; CDR, clinical dementia rating; CDT, clock drawing test; CERAD, Consortium to Establish a Registry for Alzheimer’s Disease evaluation; DRS, dementia rating scale; ESD, extended scale for dementia; HVLT-R, Hopkins verbal learning test-revised; MMSE, mini mental state examination; mMMS, modified mini-mental state examination; MTS, 37-item mental test score; NEVIP, Newcastle visual perception battery; TMT A, trail making test A; VOSP, visual object and space perception battery; WAIS, Wechsler adult intelligence scale; WISC-R, Wechsler intelligence scale for children-revised; WMS, Wechsler memory scale.

**Table 4:** Studies reporting differences in cognitive decline

**Study**	**Cognitive function**	**Impairment**	**Contrast group**	**Test**
**Olichney** ***et al*., 1998 [**[3]**]**	Total score	AD/DLB	AD	MMSE
**Kraybill** ***et al*** **l., 2005 [**[15]**]**	Total score	AD/DLB	AD and DLB	MMSE, DRS
**Nelson** ***et al*., 2009 [**[16]**]**	Total score	AD/DLB	AD and DLB	MMSE
**Heyman** ***et al*., 1999 [**[18]**]**	Delayed recall	AD	AD/DLB	CERAD
**Stavitsky** ***et al*., 2006 [**[19]**]**	Recognition	AD	DLB	HVLT-R
**Ballard** ***et al*., 1996 [**[17]**]**	Verbal fluency	DLB	AD	CAMCOG

AD, Alzheimer’s disease; AD/DLB, mixed pathology; DLB, dementia with Lewy bodies;

CAMCOG, Cambridge cognitive examination; CERAD, Consortium to Establish a Registry for Alzheimer’s Disease evaluation; DRS, dementia rating scale; HVLT-R, Hopkins verbal learning test-revised; MMSE, mini mental state examination.

**Figure 1:**
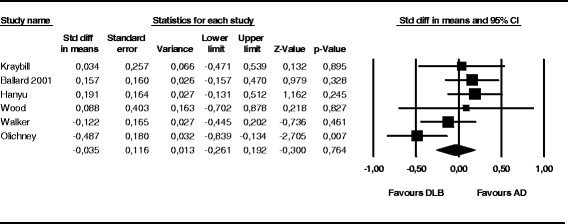
**Forrest plot of annual progression of mini-mental state examination scores.** The random-effects meta-analysis revealed an overall effect-size of −0.035 (negative sign indicates faster progression in dementia with Lewy bodies (DLB) (*P* = 0.764; 95% CI = 0.261, 0.192). AD, Alzheimer's disease.

Six studies either reported annual decline in MMSE scores, or included data enabling calculation of annual decline based on reported scores. In AD, mean annual decline was 3.3 (SD 1.7, range 1.8 to 4.9), and in DLB 3.4 (SD 1.4, range 1.8 to 5.8). One study also reported annual decline of 5.0 in AD/DLB (see Figure 1). The random-effects meta-analysis revealed an overall effect-size of −0.035 (negative sign indicates faster progression in DLB) (*P* = 0.764; 95% CI = 0.261, 0.192). *I*^2^ was 50.3, which is considered to represent moderate heterogeneity [14].

### 3.1 Cognitive domains

Six studies measured memory, and two reported differences in memory over time, both a faster decline in AD. Delayed recall was found to have a faster decline in AD compared to AD/DLB when measured with the Consortium to Establish a Registry for Alzheimer’s Disease (CERAD) evaluation, with 15% of patients with AD versus 32% of patients with AD/DLB remembering any item at the last evaluation [17]. Recognition was found to have a faster decline in AD compared to DLB as measured with Hopkins verbal learning test- revised (HVLT-R) (scores not available) [19]. Eight studies measuring language and ten studies measuring visuospatial ability reported no differences in rate of decline. Seven studies measured explicit executive functions, and one reported differences over time. In that study, verbal fluency was found to have a more rapid decline in DLB compared to AD, measured with the Cambride cognitive examination (CAMCOG) (subscores not available) [17].

### 3.2 Subgroups

Two studies [28],[30] divided patients into two groups according to high or low visuospatial functioning. In the first study, DLB patients with a low baseline score (<20) on the Wechsler intelligence scale for children-revised, block design (WISC-R) and impaired clock drawing test (CDT) had a faster decline on the dementia rating scale (DRS), compared to DLB patients with a high baseline score. In the latter study, DLB patients with a low baseline score on the Newcastle visual perception battery (NEVIP) had a faster decline in activities of daily living (ADL) than those with higher score, but no difference on any of the cognitive tests. There were no differences in the AD groups.

### 3.3 Quality assessment

The mean quality score for all the included studies was 9.4 points (SD 2.5, range 5 to 14) (see Table 5). Only two studies were rated fair or good on all quality measures [26],[27]. Three studies were rated poor on one variable, but fair and good on the others [15],[16],[22]. Mean quality scores for studies that found any differences in cognitive decline was 9.8 points (SD 2.4, range 5 to 11) compared to 9.3 points (SD 2.6, range 5 to 14) in the group with no differences (*P* = 0.335).

**Table 5:** Quality assessment results

**Study**	**Sum**	**Patients**	**Neuropsychological tests**	**Time**	**Autopsy**	**Clinical criteria**
**Williams** ***et al*., 2006 [**[27]**]**	14	3	3	3	3	2
**Johnson** ***et al*., 2005 [**[26]**]**	13	2	3	3	3	2
**Heyman** ***et al*., 1999 [**[18]**]**	11	1	3	3	3	1
**Lopez** ***et al*., 2000 [**[22]**]**	11	2	1	3	3	2
**Kraybill** ***et al*., 2005 [**[15]**]**	11	2	2	3	3	1
**Olichney** ***et al*., 1998 [**[3]**]**	11	3	1	3	3	1
**Nelson** ***et al*., 2009 [**[16]**]**	11	2	1	3	3	2
**Stern** ***et al*., 2001 [**[23]**]**	10	1	2	3	3	1
**Stavitsky** ***et al*., 2006 [**[19]**]**	10	1	3	3	1	2
**Hamilton** ***et al*., 2008 [**[28]**]**	10	1	3	1	3	2
**Helmes** ***et al*., 2003 [**[25]**]**	9	0	2	3	3	1
**Hanyu** ***et al*., 2009 [**[29]**]**	9	3	1	3	0	2
**McKeith** ***et al*., 1992 [**[20]**]**	8	1	1	3	3	0
**Ballard** ***et a*** **l., 2001 [**[24]**]**	8	3	2	0	1	2
**Walker** ***et al*., 2012 [**[31]**]**	8	3	3	0	0	2
**Wood** ***et al*., 2012 [**[30]**]**	6	0	3	0	0	3
**Ballard** ***et al*., 1998 [**[21]**]**	5	1	1	0	1	2
**Ballard** ***et al*., 1996 [**[17]**]**	5	1	2	0	0	2

### 3.4 Clinical and neuropathological diagnostic criteria

There were no systematical differences in clinical or neuropathological criteria between studies that found differences in cognitive decline and those who did not (see Table 6). Of 18 included studies, 16 (89%) used National Institute of Neurological and Communication Disorders and Stroke/Alzheimer’s Disease and Related Disorders Association (NINCDS/ADRDA) or CERAD clinical criteria for AD and 12 (67%) used DLB consensus criteria, only one of them used the revised criteria from 2005. To diagnose AD neuropathologically, mainly CERAD neuropathological criteria for the diagnosis of AD and neuropathological DLB consensus criteria from 1996 were used. A diagnosis of mixed AD/DLB was made, if in addition to the Alzheimer’s pathology the characteristic Lewy bodies were found in subcortical and cortical areas. Eleven studies (61%) used autopsy-confirmed diagnosis on all patients. In three studies (17%), some of the diagnoses were autopsy-confirmed. In four studies (22%) autopsy was not performed. One of the studies used ^123^I-FP-CIT-SPECT only as a method of verifying of clinical diagnosis [31].

**Table 6:** Clinical and neuropathological criteria

**Study**	**Sample**	**Database**	**Neuropathological criteria**	**Autopsy**	**Dementia criteria**
**McKeith** ***et al*., 1992 [**[20]**]**	AD 37	Newcastle, UK	AD: plaque/tangle quantification, H + E, CFV, Loyez, Palmgren.	All	DLB: proposed consensus (1992)
	SDLT 21				
			LB: H + E, pholxine, erythrosin		
**Ballard** ***et al*., 1996 [**[17]**]**	AD 53	West Midlands and Bristol, UK		0	AD: NINCDS/ADRDA (1984)
	SDLT 7				DLB: McKeith, operational criteria for senile dementia of Lewy body type (1992)
	VaD 14				
**Ballard et al., 1998 [**[21]**]**	AD 30	Newcastle General Hospital, UK	AD: CERAD, plaque – Braunmuhl stain, tangle – modified Palmgren	19	AD: NINCDS/ADRDA (1984)
	DLB 42				DLB: McKeith, operational criteria for senile dementia of Lewy body type (1992)
			LB: consensus criteria (1996), ubiquitin, anti-tau2, anti-Alz50, anti-AT8 to detect and distinguish cortical LB		
**Olichney** ***et a*** **l., 1998 [**[3]**]**	AD 148	Cohort from:	AD: CERAD, ADRC	All	AD: NINCDS/ADRDA (1984),
	LBV 40	Univeristy of California, San Diego Alzheimer’s Disease Research Center, USA;	LB: ubiquitin, H + E (brainstem, cerebral cortex)		DSM-III for dementia
		CERAD centers, multinational			
**Heyman** ***et al*., 1999 [**[18]**]**	AD 74	Subjects with premortem diagnosis of probable and possible AD from 24 centers participating in CERAD, 1986 to 1995, USA	AD: CERAD	All	AD: NINCDS/ADRDA (1984)
	AD/LBV 27		LB: consensus criteria (1996), modified (brainstem, limbic/transitional and noecortical).		
**Lopez** ***et al*., 2000 [**[22]**]**	AD 98	University of Pittsburg 1983 to 1998, USA	AD: CERAD, NIA-RI	All	AD: NINCDS/ADRDA (1984)
	AD/DLB 44		LB: H + E, ubiqutin (SN, neocortex, limbic areas)		DLB: consensus criteria (1996)
**Stern** ***et al*. 2001 [**[23]**]**	AD 32	From cohort of 236 patients with probable AD	AD: CERAD	All	AD: NINCDS/ADRDA (1984)
	LBV 19		LB: semi quantitative ubiquitin (SN, hippocampus, cingulate gyrus, insula cortex)		
		Recruited:			
		Columbia University College, New York, USA			
		Johns Hopkins University, Baltimore, USA			
		Massachusetts General Hospital, Boston, USA			
**Ballard** ***et al*., 2001 [**[24]**]**	AD 101	Cohort of 227 patients	AD: CERAD, plaque - Braunmuhl stain, tangle - modified Palmgren	50	AD: NINCDS/ADRDA (1984)
	DLB 64	Institute of the Health of the Elderly (IHE), Newcastle, UK			DLB: consensus criteria (1996)
	VaD 38		LB: consensus criteria (1996), ubiquitin, anti-tau2, anti-Alz50, anti-AT8 to detect and distinguish cortical LB		
**Helmes** ***et al*., 2003 [**[25]**]**	AD 15	University of Western Ontario Dementia Study, Canada	No criteria are referred to. Only referred to LB staining methods (Bielschovsky, anti-ubiquitin, anti-synuclein).	All	Not specified.
	AD/DLB 8				
	DLB 7				
**Johnson** ***et al*., 2005 [**[26]**]**	AD 66	Washington University, from 1979, USA	AD: NIA-RI quantification of diffuse and neuritic depositions in 10 cortical regions	All	AD: NINCDS/ADRDA (1984)
	AD/DLB 57				DLB: consensus criteria (1996) or McKeith, operational criteria for senile dementia of Lewy body type (1992)
	DLB 9		LB: synuclein		
**Kraybill** ***et al*., 2005 [**[15]**]**	AD 48	Cohort from University of Washington/Group Health Cooperative Alzheimer’s Disease Patient Registry, USA	AD: CERAD, Braak stages > IV	All	AD: NINCDS/ADRDA (1984)
	AD/LBP 65		LB/AD: AD + synuclein (amygdala, SN)		DLB: missing criteria because study was started before the consensus criteria for DLB was established.
	LBP 22				
			LB: Braak stages < III, synuclein (amygdala, SN)		
**Stavitsky** ***et a*** **l., 2006 [**[19]**]**	AD 55	Cohort of the Predictors Study, 1997:	AD: CERAD	12	AD: NINCDS/ADRDA (1984)
	DLB 28		LB: semi quantitative ubiquitin (hippocampus, cingulate gyrus, insula cortex)		DLB: consensus criteria (1996)
		Columbia University			
		Johns Hopkins University,			
		Massachusetts General Hospital, USA			
**Williams** ***et al*., 2006 [**[27]**]**	AD 252	Cohort from Washington University, USA	AD: NIA-RI quantification of diffuse and neuritic depositions in 10 cortical regions	All	AD: NINCDS/ADRDA (1984)
	DLB 63				DLB: consensus criteria (1996)
			LB: synuclein		
**Hamilton** ***et al*., 2008 [**[28]**]**	AD 44	University of California, Alzheimer’s disease center San Diego, 1985 to 2002, USA	AD: modified Braak staging, NIA-RI (1997) and CERAD (1991)	All	AD: NIA-RI and CERAD (1988)
	DLB 22				DLB: consensus criteria (1996)
			LB: H + E, ubiquitin (1996) synuclein (2005)		
**Hanyu** ***et al*., 2009 [**[29]**]**	AD 111	Memory Clinic of Tokyo Medical University, 2000 to 2006, Japan		0	AD: NINCDS/ADRDA (1984)
	DLB 56				DLB: consensus criteria (1996)
**Nelson** ***et al*., 2009 [**[16]**]**	AD 107	National Alzheimer’s Coordinating Center (NACC) Registry - 31 AD centers in USA,	AD: NIA-RI	All	AD: CERAD (1988)
	AD/DLB 27	University of Kentucky Alzheimer’s Disease Center, USA	LB: Braak staging and CERAD		DLB: consensus criteria (1996)
	DLB 9				
**Wood** ***et a*** **l., 2012 [**[30]**]**	AD 16	Newcastle University, UK		0	AD: NINCDS/ADRDA (1984)
	DLB 12				DLB: consensus criteria (2005) or (1996)
**Walker** ***et al*. 2012 [**[31]**]**	AD 100	40 European sites	^123^I-FTP-SPECT as verifying method	0	AD: NINCDS/ADRDA (1984)
	DLB 58				DLB: consensus criteria (1996)

AD, Alzheimer’s disease; ADRC, Alzheimer’s Disease Research Center; CERAD, The Consortium to Establish a Registry for Alzheimer's Disease; CFV, creasyl fast violet; DLB, dementia with Lewy bodies; H + E, hematoxylin and eosin staining; I-FTP-SPECT, ioflupane single-photon emission computed tomography; LB Lewy body; LBV, Lewy body variant; LBP, Lewy body pathology; NIA-RI, National Institute on Aging-Reagan; NINCDS/ADRDA, National Institute of Neurological and Communication Disorders and Stroke/Alzheimer’s Disease and Related Disorders Association; SDLT, senile dementia of Lewy body type; SN, substantia nigra.

## 4 Discussion

In the 18 studies included in this review, no consistent faster rate of decline in DLB as compared to AD on cognitive screening tests was found. When combining studies that used MMSE, the most frequently used scale, a meta-analysis revealed no difference in the annual rate of cognitive decline. There were mixed findings on decline in specific cognitive domains. Two of six studies of memory found a more rapid decline in AD. Only one of seven studies of executive function found a more rapid decline in DLB, and differences in visuospatial or language tests were not found. The hypothesis of a more rapid cognitive decline in autopsied patients with both AD and DLB pathology was supported in three studies. However, findings were inconsistent and other studies did not find differences.

Differences in methods such as selection criteria, design, neuropsychological tests, dementia severity, diagnostic procedures and criteria can explain the diverse findings and lack of firm conclusions. However, quality assessment did not reveal any systematic differences between studies with high or low quality scores. There were large differences in sample sizes (n = 28 to 315), and the studies that could not be included in the meta-analysis or used other tests than MMSE, thus, may have had varying statistical power to detect significant differences between groups. To be able to compare the overall results and draw some general conclusions it would have been ideal that uniform diagnostic criteria had been used in all the studies. Some of the studies initially included patients with a clinical diagnosis of AD only, where analyses were based on autopsy diagnosis which included both AD and DLB.

A common weakness in the included studies was the choice of neuropsychological measures. When studying cognitive decline over time, cognitive tests that are designed for a specific cognitive domain are required. Screening tests or batteries that use a total score only, often designed for purposes other than research are less suitable. In this review, the MMSE was the most used test, either alone, or in combination with others. The MMSE may not be an optimal measure, especially when using only the total score and not separate subscores for different cognitive domains, as AD and DLB have different cognitive profiles at onset [32]. This difference in cognitive profile leads to difficulties in choosing an optimal cognitive screening instrument to compare AD and DLB. The MMSE is heavily based on memory and language and is thus more sensitive to changes in AD than in DLB [33]. DLB is associated with a more severe visuospatial deficit than AD [32],[34], but only 1 of 30 points on the MMSE comes from a measure of visuospatial functioning. MMSE may also be less than optimal because of the ceiling and floor effect [35], which refers to a test being too easy or too difficult to discriminate below or above a certain point, which is a common problem when testing people with dementia. In one of the reviewed studies the children’s version of the Wechsler intelligence scale was used to avoid this. The test then lacks age adjusted norms, but it gains a wider range in scores, and therefore can monitor the cognitive decline over a longer period of time. Studies differed also with regard to the time period of observation, from 1 to 20 years. In studies with short follow-up periods, the MMSE may not be a reliable measure, as Clark, Sheppard, Fillenbaum *et al*. (1999) [36] have argued that MMSE registrations need to be separated by at least three years in order to be a reliable measure of cognitive decline in AD.

Only few studies investigated, or reported, subgroups with different cognitive profiles in DLB. It could be due to a low number of cases in several studies, and subsequent low statistical power. People die from dementia or reach an endpoint where they are not capable of performing cognitive tests, and therefore in several studies there was a lower number of patients towards the end of the study. This is challenging when performing statistical analysis. Our search did not cover the issue of subgroups with different cognitive profiles thoroughly, as we only included studies comparing DLB with AD, and not studies describing cognitive decline in DLB and potential subgroups alone. However, there are some data that support the hypothesis that there are subgroups in DLB with different cognitive profiles, and subgroups with poor initial visuospatial function may have a more rapid decline than DLB with good visuospatial function [28].

Due to overlapping symptoms, it can be difficult to determine the correct diagnosis ante mortem between the pure form of AD, mixed AD/DLB and the pure form of DLB. Because clinical criteria cannot distinguish with certainty the individual pathology, the gold standard for validating the clinical assessment is neuropathological diagnosis. Clinical criteria may have a low sensitivity in particular for DLB, which could have been a source of bias in studies that did not include a neuropathological validation of the diagnosis. However, dementia is a clinical diagnosis and both AD and DLB pathology can be found also in cognitively normal elderly subjects. In one study with autopsy, 50% of cases with widespread α-synucleinopathy did not show any clinical signs of dementia [37].

In most studies with autopsy, consensus neuropathological criteria were used. Even though not all included studies used consistent and the same neuropathological methods and criteria, and many also used varying combinations, use of post-mortem verification at least increases the validity of the clinical diagnosis.

It is also important to mention that the sensitivity for detecting Lewy bodies has increased with anti-ubiquitin immunostaining, where tau-positive samples indicate Alzheimer’s pathology. Anti-α-synuclein immunostaining has been incorporated in the assessment, which is most sensitive for Lewy body pathology [2]. Thus, the neuropathological identification of cases may have been less accurate before the new methods were established, and more reliable staging strategies have been developed [38].

A complicating issue is the frequent occurrence of mixed pathology [39], and to underline the complexity of dementia and its pathology, at least four distinct pathological phenotypes have been identified between AD and DLB [40]. According to Schneider *et al*. (2012) [7], the locus of neuropathology is associated with a faster decline in cognition. A neocortical type of Lewy body pathology is associated with increased odds of dementia and a faster decline in episodic, semantic and working memory. The limbic-type is more associated with more rapid decline in visuospatial function. Olichney *et al*. (1998) [3], concluded that patients with Lewy body variant decline faster than patients with Alzheimer’s disease. This statement has often been used with reference to rapid progression in DLB, but it actually refers to an AD variant with Lewy body pathology, not to pure DLB. It should be emphasized that it is still uncertain whether AD and DLB are two independent pathologies that may coexist, or the pathologies are related, or one of them is a consequence of the other.

## 5 Conclusion

Only 6 of the 18 included studies in this review found some differences in cognitive decline between DLB and AD over time, and only one of them found a faster decline in DLB. It is difficult to draw firm conclusions based on available studies, since the results are contradictory. Future studies will need to apply recent diagnostic criteria, as well as extensive diagnostic evaluation and autopsy to confirm the diagnosis. Studies with large enough samples, adapted cognitive tests, more than one year of follow up and multivariate statistical analysis are also needed. Inclusion of mild cognitive impairment patients, with subclinical manifestations and an increased risk of developing DLB (for example, who present rapid eye-movement (REM) sleep behavior disorder) could also strengthen the studies. Our final conclusion is that the studies in this review support neither the hypothesis of a faster cognitive decline in DLB, nor in AD.

## Abbreviations

AD: Alzheimer’s disease

ADL: activities of daily living

CAMCOG: Cambride cognitive examination

CDT: clock drawing test

CERAD: Consortium to Establish a Registry for Alzheimer’s Disease evaluation

DLB: dementia with Lewy bodies

DRS: dementia rating scale

HVLT-R: Hopkins verbal learning test-revised

MMSE: mini mental state examination

NEVIP: Newcastle visual perception battery

NINCDS/ADRDA: National Institute of Neurological and Communication Disorders and Stroke/Alzheimer’s Disease and Related Disorders Association

SPECT: ioflupane single-photon emission computed tomotgraphy

WISC-R: Wechsler intelligence scale for children-revised

## Competing interests

Dag Aarsland has received research support and honoraria from H Lundbeck, Novartis Pharmaceuticals and GE Health. None of the other authors have competing interests.

## Authors’ contributions

MHB and LJC have made the conception and design, data acquisition, analysis and interpretation of data, and drafted the manuscript. AR, MJH, CJ, KB and DA have contributed to the analysis and interpretation of data, and revised the manuscript critically for important intellectual content. KB also performed the meta-analysis. All authors have read and approved the final version of the manuscript.
